# Experimental Study on the Mechanical Properties and Influencing Factors of Glass Fiber-Reinforced Permeable Concrete

**DOI:** 10.3390/ma16175970

**Published:** 2023-08-31

**Authors:** Lina Xu, Xu Ding, Lei Niu, Zhanfang Huang, Shuang Sun

**Affiliations:** 1 School of Transportation Science and Engineering, Jilin Jianzhu University, Changchun 130118, China; xulina@jlju.edu.cn; 2 School of Civil Engineering, Jilin Jianzhu University, Changchun 130118, China; dingxu0902@outlook.com (X.D.); niulei2016@163.com (L.N.); 3 School of Architectural Engineering and Space Information, Shandong University of Technology, Zibo 255049, China; huangzhanfang@163.com

**Keywords:** glass fiber reinforced permeable concrete, water permeability coefficient, mechanical properties, fiber content, fiber length

## Abstract

In order to improve the mechanical properties and deformation characteristics of permeable concrete, glass fiber was added to this type of concrete. Based on an unconfined compressive strength test, non-contact full-field strain measurement system, and scanning electron microscopy test, the effects of aggregate particle composition, shaking time, fly ash content, fiber length, and fiber content on the strength and permeability of permeable concrete were studied. The results show that the strength and water permeability of permeable concrete are negatively correlated with an increase in shaking time. When the aggregate particle size is 5–10 mm, the permeable concrete has both good strength and permeability. Proper incorporation of fly ash improves the compactness inside the structure. The influence of different lengths of glass fiber on the strength of permeable concrete first increases and then decreases, and the permeable property decreases. With the same fiber length, the strength increases first and then decreases with an increase in the content, while the porosity and water permeability coefficient decrease. Under the test conditions, when the length of glass fiber is 6 mm, and the dosage is 2 kg/m^3^, the strength performance of permeable concrete is the best, and the permeability effect is good at the same time.

## 1. Introduction

Today’s global trends are changing rapidly, and research related to cementitious composites tends to be green, environmentally friendly, and sustainable [1,2]. In the field of urban construction, the introduction of new concepts such as the sponge city and resilient city has added a new development impetus to road infrastructure construction and promoted the green development of transportation infrastructure.

The construction and promotion of “sponge cities” is an important development in the field of urban construction. Permeable concrete is mainly used in urban construction because of its characteristics of high strength, green environment, and good drainage effect [3,4]. Domestic and foreign scholars have found that aggregate particle size and type [5,6], cement–bone ratio [7], auxiliary cementing material inclusion ratio [8], shaking method [9], and other factors have prominent effects on various properties of permeable concrete. Lei et al. [10] found through their study that various modification methods have different effects on the road performance of asphalt mixtures, and soaking RCA with slag powder and silane coupling agents was prominent among them. Li et al. [11,12] established the relationship between porosity and coating thickness by measuring parameters, such as the shape index and grading of coarse aggregate and verified the relationship between the thickness of aggregate coated by cement paste and porosity by using a mathematical model. Carmichael et al. [13], in their conclusions from tests on the partial replacement of cement by nano-fly ash, found that a 1:6 mix, a 0.34 water–binder ratio, and a 40% replacement fraction of cement through nano-fly ash were the optimum among the other mixing compositions. Wu et al. [14] studied the law of the influence of molding method and sand rate on strength and water permeability and found that adding fine aggregate could not only improve mechanical properties but also ensure the water permeability effect. Xu et al. [15], based on the mechanics and permeable properties of permeable concrete, used Design Expert software (https://www.statease.com) to optimize the mix ratio parameters of permeable concrete, such as cement dosage, water-cement ratio, aggregate gradation, and particle size and concluded that the water–cement ratio had no obvious effect on the performance indexes of permeable concrete. Cheng et al. [16] proved that mineral powder and fly ash had a certain deterioration effect on the strength of permeable concrete by adding them to this type of concrete, but it was also found that they improved its water permeability. Gong et al. [17] illustrated through tests that when using pressure molding methods, the strength of permeable concrete increases and then decreases with increasing pressure, while when using vibratory molding, the strength of permeable concrete shows an increasing trend with increasing shaking time. By studying the effect of two forming methods on porous concrete, Sheng et al. [18] confirmed that the strength of porous concrete is higher with vibratory forming, while the flatness is better with vibration-free forming.

Permeable concrete has good characteristics of water permeability, but it has certain deficiencies in strength and durability. Therefore, domestic and foreign scholars have mixed polypropylene fiber, basalt fiber, and carbon fiber into permeable concrete [19,20,21] to improve the mechanical properties and long-term service performance of permeable concrete [22]. Meeju et al. [23] investigated the use of polyethylene terephthalate (rPET) fibers for strengthening construction materials and confirmed that these fibers can be used in coastal structures that require impact-resistant non-metallic materials. Akand et al. [24] added 19 mm treated polypropylene fiber into permeable concrete as a reinforcement material, proving that chemical treatment could improve the bonding effect between the fiber and the slurry, so the strength of permeable concrete could be significantly improved. Liu et al. [25], through a series of tests, confirmed that the incorporation amount of rigid polypropylene fiber had a significant impact on the durability of permeable concrete. Meng et al. [26] found that adding a geogrid to permeable concrete significantly improved the performance of this type of concrete and reduced porosity.

At present, there are relatively few studies on short-cut glass fiber-reinforced permeable concrete. Based on this, this paper adds glass fiber with different lengths and contents to permeable concrete to study the forming method, aggregate particle composition, and fly ash content of permeable concrete and studies the influence of the length and content of glass fiber on various performance indexes of permeable concrete. The optimum fiber length and incorporation ratio were obtained, the fiber reinforcement mechanism was revealed, and a theoretical reference was provided for its application in road engineering.

## 2. Test Materials and Test Process

### 2.1. Test Materials

**Figure 1:**
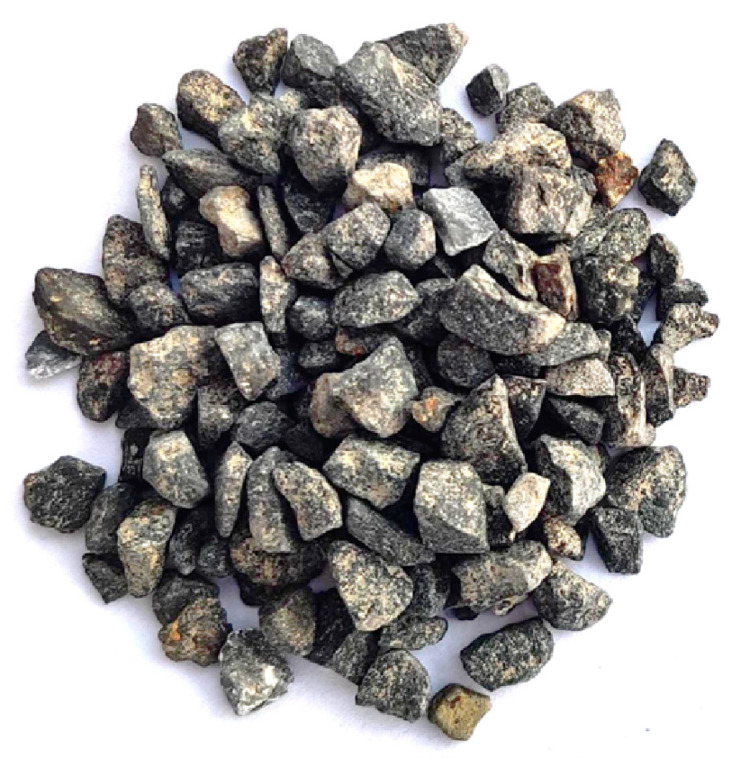
Aggregate photograph.

**Figure 2:**
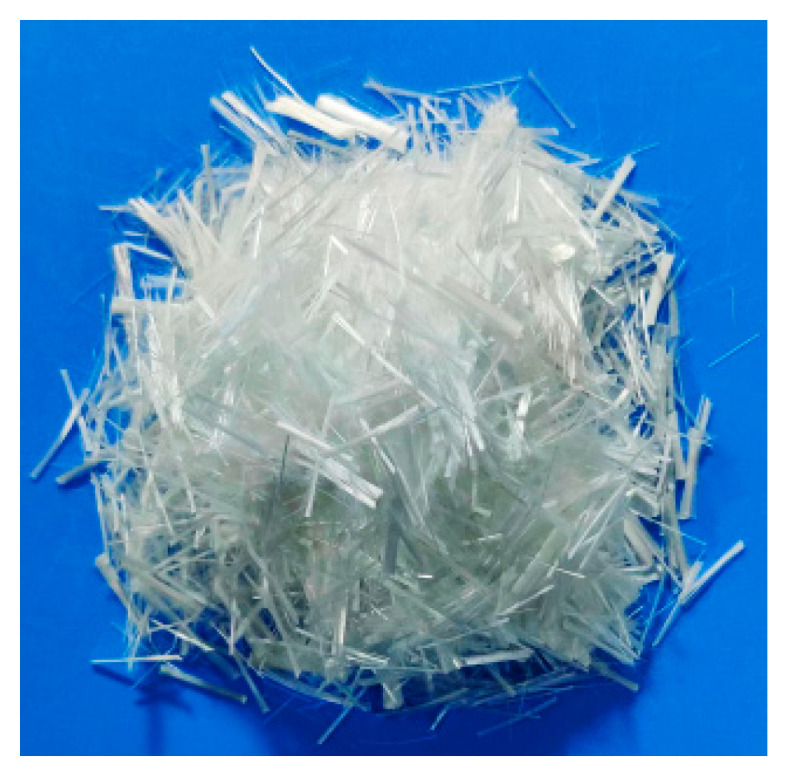
Glass fiber photograph.

The cement used in this test is ordinary Portland cement with a strength grade of P.O 42.5. The physical properties are shown in Table 1. The national standard first-class fly ash is selected as the auxiliary cementing material, and the chemical composition is shown in Table 2. For the Guangdong province -produced aggregate (Figure 1), there is an aggregate gradation of 3–5 mm, 5–10 mm, and 10–15 mm, and the basic aggregate performance indicators are shown in Table 3. Polycarboxylic acid superplasticizer was selected for this study. The reinforced material used is short-cut ordinary glass fiber (Figure 2), with a diameter of about 0.012 mm, a tensile strength of about 1800 MPa, tensile limit elongation of about 4%, and good dispersion.

### 2.2. Experimental Mix Ratio Design

In this test, the volume method was used to prepare the specimens. According to the Technical Specification for Pervious Cement Concrete Pavement (CJJ/T135-2009) [27], the design porosity was 20%, the water-binder ratio was 0.30, and the mix ratio design was calculated according to Equation (1). The mix ratio design parameters of the ordinary permeable concrete test are shown in Table 4. The test scheme and mix ratio design parameters of glass fiber permeable concrete are shown in Table 5.

(1) $$\frac{m_{c}}{\rho_{c}}+\frac{m_{f}}{\rho_{f}}+\frac{m_{g}}{\rho_{g}}+\frac{m_{w}}{\rho_{w}}+\frac{m_{a}}{\rho_{a}}+P=1$$

where *m* is the mass of various materials; *ρ* is the apparent density of the material; *P* is the target porosity; *c* is the cement; *f* is the admixture; *g* is the coarse aggregate; *w* is water; and *a* is a water-reducing agent.

### 2.3. Sample Preparation

**Figure 3:**
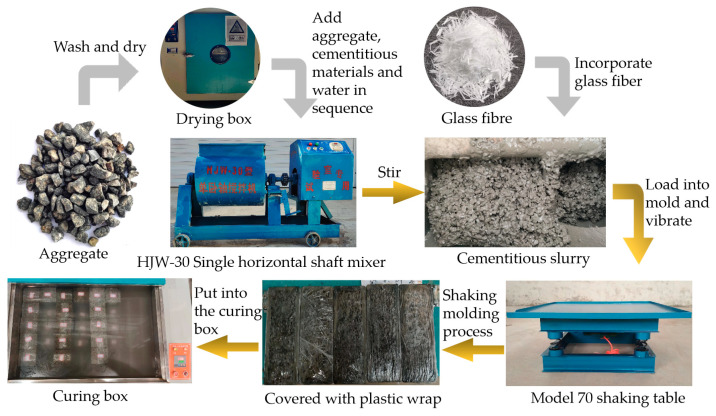
Preparation flow chart.

The specimens used in the test were prepared according to Technical Specification for Pervious Cement Concrete Pavement (CJJ/T135-2009) [27], and the specimen size was 100 mm × 100 mm × 100 mm. The specific process is described below, and the steps are shown in Figure 3.

1. All of the aggregates used in the test were cleaned, with the aim of removing surface sand and impurities, dried, and sieved through a drying oven to obtain a single size.
2. An HJW-30 single horizontal shaft mixer was used to mix the cement and auxiliary cementitious materials for 30 s first, and then, the aggregate was added with continual mixing for 90 s to ensure that the materials were fully mixed.
3. After the dry material was mixed evenly, the water-reducing agent and water were added, and mixing continued for 90 s.
4. The glass fibers were soaked in 1.5% polycarboxylic acid water-reducing agent solution for 5 min, and then, the fibers were taken out and dried. The dried fibers were divided into 3–5 equal parts to achieve the best possible dispersion of the glass short-cut fibers. In the mixing process of step 3, one part of the glass fiber was evenly sprinkled in the mixture, and the fiber was added while mixing and then the step was repeated until all the glass fibers were mixed in to ensure that the fibers were well dispersed in the mixture.
5. Shaking and molding test was performed using a Tianjin Qingda Testing Instrument Manufacturing Co., Ltd. (Tianjin, China) product of a 70-type shaker with a vibration frequency of 2860 times/min. The mold was placed on the shaking table and a 50 mm-high baffle was placed around the outer edge of the mold. The finished slurry was then injected into the mold up to 2/3 of the baffle, and the switch was turned on to shake the mixture for the desired amount of time. After shaking, the baffle was removed. A scraper was used to gently scrape off the excess slurry and flatten the top surface.
6. After, the specimen was poured and covered with plastic film to prevent water loss. Then, it was left for three days at room temperature before the specimen was removed from the mold. After the specimens were removed from the molds, they were placed in a curing box at a constant temperature of 20 °C until the required age for the test.

### 2.4. Test Methods

#### 2.4.1. Unconfined Compressive Strength Test

**Figure 4:**
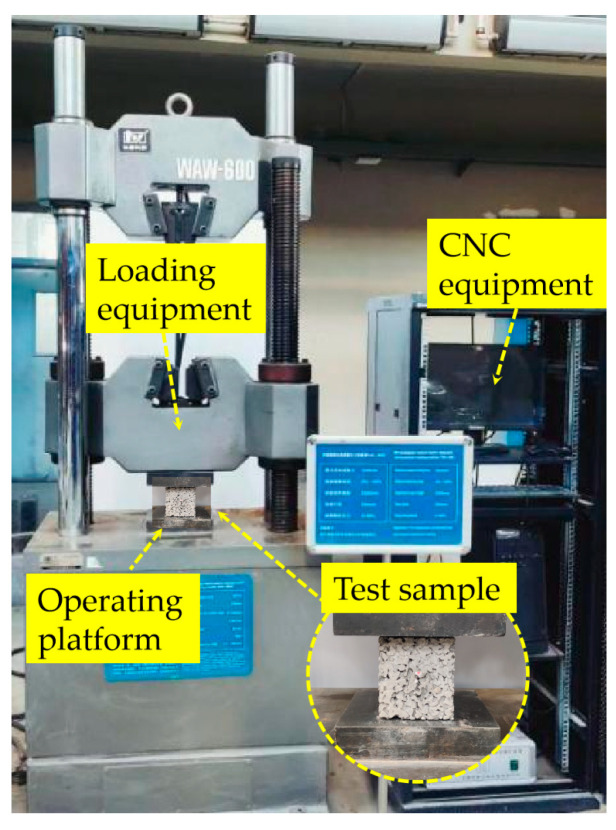
Microcomputer servo pressure testing machine.

The mechanical property test was carried out in accordance with the test method in the Standard for Test Methods of Concrete Physical and Mechanical Properties (GB/T 50081-2019) [28], and a microcomputer servo pressure testing machine (WAW-600KN) was selected to carry out the test, with a loading speed of 0.1 mm/s, as shown in Figure 4. The data collection of unconfined compressive strength was completed by the automatic collection system matched with a WAW-600 universal testing machine. The data collection process includes load and displacement. 

#### 2.4.2. Porosity Measurement

**Figure 5:**
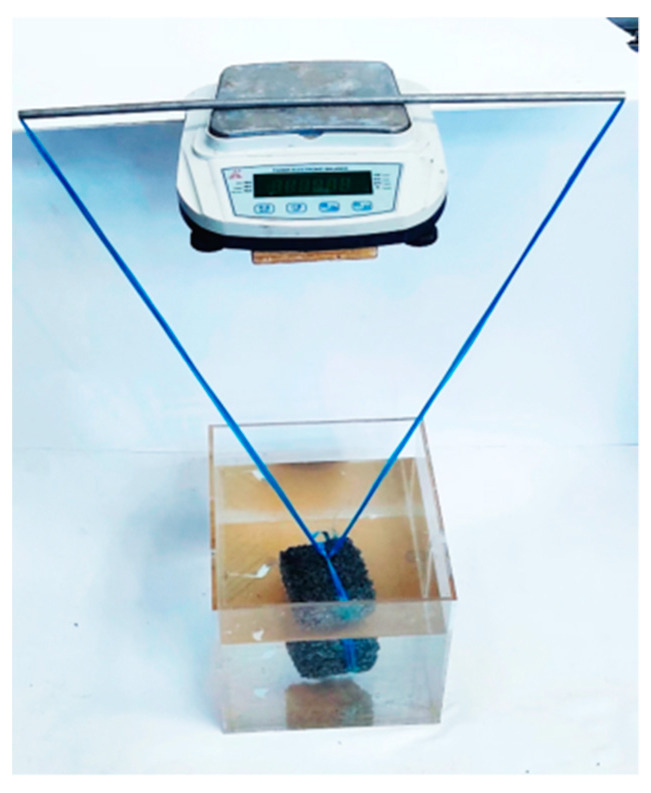
Porosity measurement equipment.

In this paper, the porosity of permeable concrete was tested by the weight method, which is based on the principle of using the mass difference between dried and saturated specimens to characterize the magnitude of the buoyancy value of the specimen after the pores are filled with water. The pore space is divided into connected, semi-connected, and closed pores. The connected pores play a major role in permeable concrete, and the semi-connected pores are in a relatively stagnant state during the drainage stage, which is ineffective for the drainage effect. Therefore, it is the effective porosity, i.e., the ratio of effective pore volume to total volume, that determines the water permeability coefficient of permeable concrete. The test method is shown in Figure 5.

The permeable concrete was soaked in water for 24 h to saturate the mass M1 of the specimen. The specimen was taken out, wiped dry, and placed in the air to dry, and after the mass was constant (about 24 h, the exact time depends on the indoor and outdoor temperatures), the mass of the specimen in the air was weighed M2, and Vt is the volume of the permeable concrete specimen. The open porosity P of permeable concrete can be calculated according to Equation (2):

(2) $$P=\left[1-\left(\frac{M_{2}-M_{1}}{\rho_{1}V_{t}}\right)\right]\times 100\%$$

where *P* is the porosity of the specimen, %; *M*_1_ is the mass in water of the specimen, g; *M*_2_ is the dry mass of the specimen, g; *ρ*_1_ is the density of water, g/cm^3^; and *V*_t_ is the volume of the test block, cm^3^.

#### 2.4.3. Measurement of Water Permeability Coefficient

**Figure 6:**
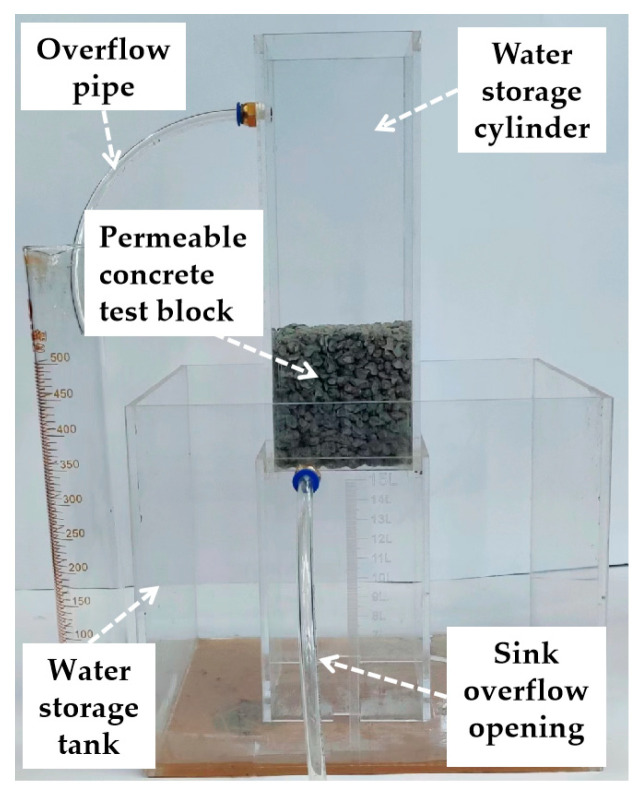
Water permeability coefficient measuring equipment.

The water permeability coefficient is an important test index of permeable concrete. This paper followed the Chinese standard specification “Technical specification for pervious cement concrete pavement” CJJ/T 135-2009 [27] to test the water permeability coefficient of permeable concrete. The test equipment used is shown in Figure 6.

In the process of water injection, the flow rate of water injection is constantly adjusted to ensure that the water level difference “H” is constant during the test time so as to ensure that the pressure on the upper surface of the test block is constant. The permeability factor is calculated in accordance with Equation (3):

(3) $$K_{T}=\frac{QL}{AH\Delta t}$$

where *K_T_* is the water permeability coefficient of the specimen, mm/s; *Q* is the amount of water seepage within the specified time, mm^3^; *L* is the thickness of the specimen, mm; *A* is the surface area of the top surface of the specimen, mm^2^; *H* is the water level difference, mm; and Δt is the water flow time.

#### 2.4.4. Scanning Electron Microscopy

**Figure 7:**
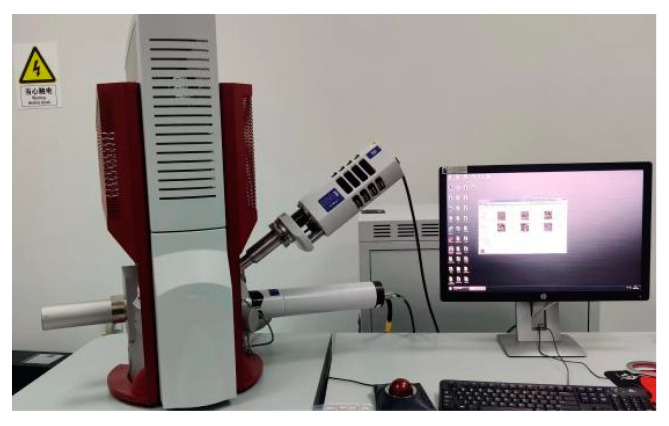
Scanning electron microscope.

A QUANTA 200 scanning electron microscope (SEM) was used to observe the solidification and hardening morphology of the cementing material between aggregates and the binding state of the fiber and cementing layer, as displayed in Figure 7.

## 3. Results and Discussion

### 3.1. Influence of Shaking Time on the Performance of Permeable Concrete

**Figure 8:**
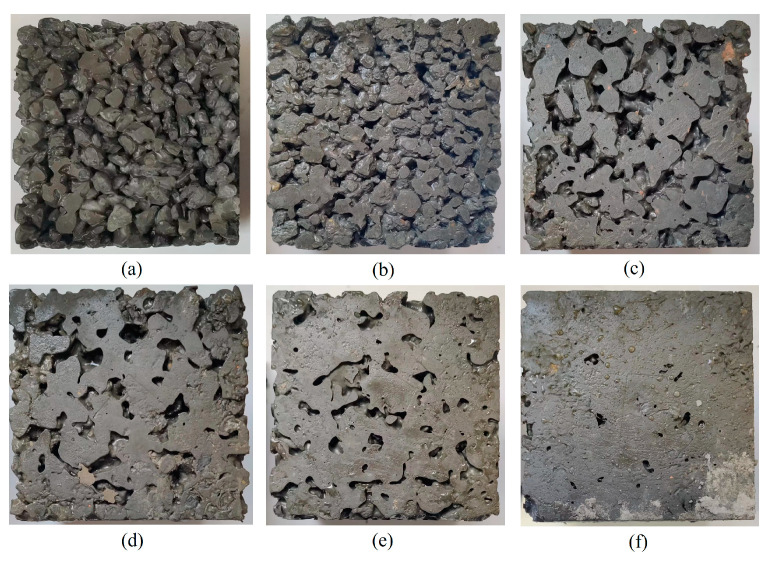
Photos of permeable concrete at different shaking times: (**a**) 3 s; (**b**) 5 s; (**c**) 10 s; (**d**) 15 s; (**e**) 20 s; and (**f**) 25 s.

Figure 8 shows photos of the bottom surface of the permeable concrete test block under different shaking times. As can be seen from the figure, the slurry sealing phenomenon at the bottom of the test block becomes more and more serious with the increase in shaking time. When the shaking time was 3 s, there was no slurry sealing phenomenon at the bottom of the test block. At 5 s, slurry aggregation appeared at the bottom of the test block. The slurry sealing phenomenon appeared at 10 s. At 15 s, there was an obvious slurry-sealing phenomenon. At 20 s, the bottom of the test block is basically completely sealed. At 25 s, the bottom of the test block is completely sealed, and the bottom is dense.

**Figure 9:**
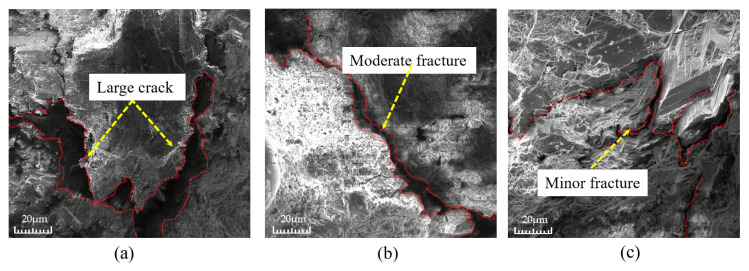
Micro-morphology of cracks between aggregates and cementation layers at different shaking times: (**a**) 5 s; (**b**) 10 s; and (**c**) 15 s.

**Figure 10:**
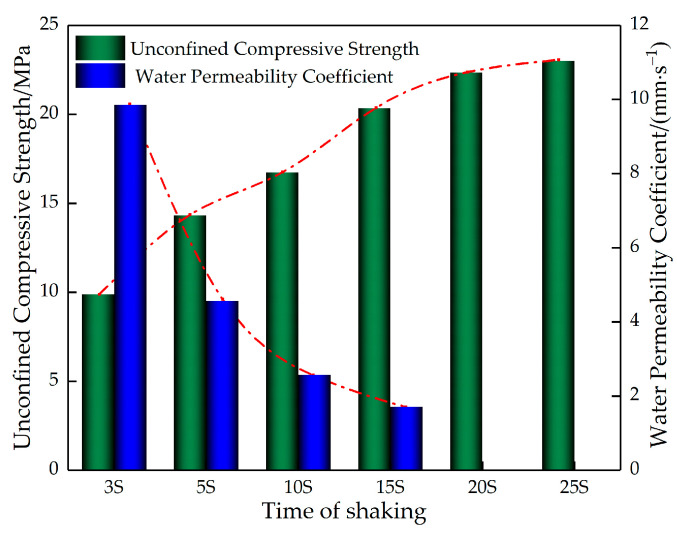
Relationship between the strength and permeable coefficient of permeable concrete.

Figure 9 shows the microscopic morphology of the aggregate wrapped by slurry at the bottom of the permeable concrete test block under different shaking times. As can be seen from Figure 9, the crack between the cement paste and aggregate is larger when the shaking time is 5 s, it is the second largest when the shaking time is 10 s, and the gap between the two is the smallest when the shaking time is 15 s. From this, it can be seen that, with the increase in the shaking time, the gap between the cement paste and the aggregate shows a tendency to decrease. Figure 10 shows the relationship between the strength and water permeability coefficient of the permeable concrete under different shaking times. Figure 10 indicates that, with an increase in shaking time, the water permeability coefficient is negatively correlated with the shaking time, while the strength is positively correlated with the shaking time. This is because the shaking process causes the aggregate to be packed tightly, forming good mechanical meshing between the aggregates. At the same time, the cement slurry wrapped around it falls under the action of gravity, and the packing density between the slurry and the aggregate is improved. In addition, most of the cement slurry is deposited to fill the pores at the bottom, resulting in a phenomenon of “scarce upper slurry and accumulation of lower slurry” [14]. Therefore, when the side of the test block is used as the top surface under pressure, the overall strength is highlighted due to the high density of the bottom, but the permeability is reduced because the connected pores are blocked.

### 3.2. Influence of Fly Ash Content on Permeable Concrete Performance

**Figure 11:**
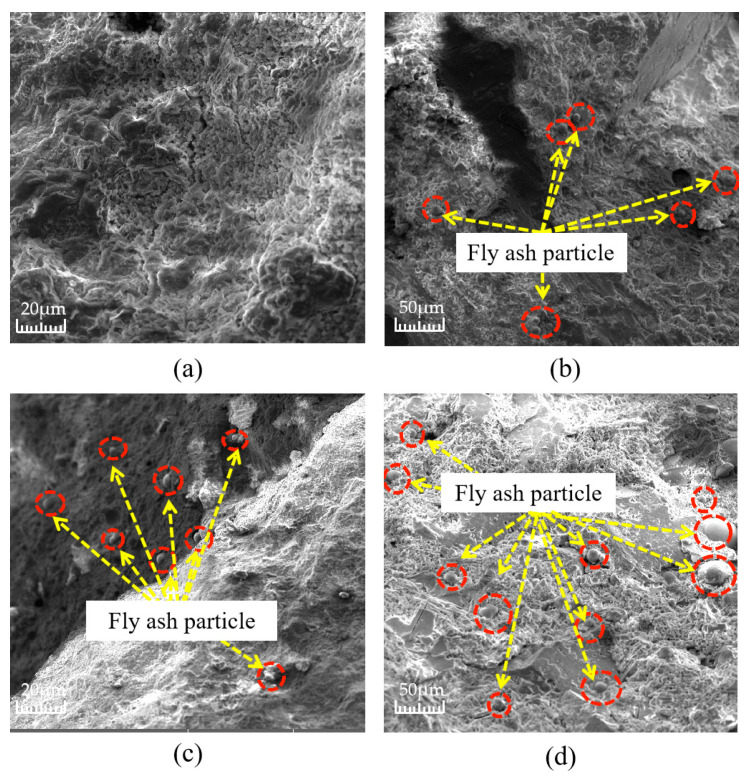
The distribution of fly ash in the cemented layers with different fly ash content: (**a**) fly ash content 0%; (**b**) fly ash content 5%; (**c**) fly ash content 7.5%; and (**d**) fly ash content 10%.

**Figure 12:**
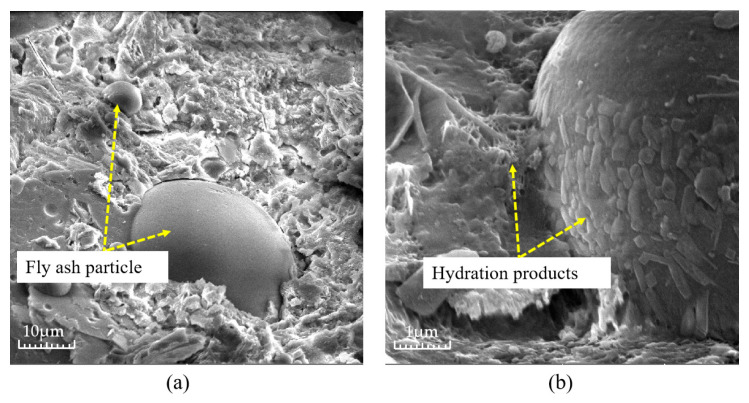
Microstructure of fly ash in the cemented layer: (**a**) 10 μm and (**b**) 1 μm.

Fly ash is one of the most common mineral fillers, the effectiveness of which has been confirmed by practical experience [29,30]. Fly ash actively affects the formation of the structure of composite building materials at all stages of hydration and the structure formation of cement systems, that is, the sequential transition from the coagulation structure to the formation of a spatial crystal framework [31]. Figure 11 shows the distribution of fly ash inside the test block under different fly ash content values, and Figure 12 shows the microscopic photos of a combination of fly ash and a cement base. As can be seen in Figure 11, with the increase in fly ash dosage, the fine particles of fly ash have a wider distribution inside the specimen slurry, and the cement slurry is denser, while the dosage of fly ash improves the fluidity of the cement slurry, which makes the cement slurry better encapsulated, and has a high degree of workability and is easier to mix. As can be seen in Figure 12, due to the active effect of the addition of fly ash, the gap between the cement and fly ash is shortened, and the hydration products of cement closely adhere to the surface of fly ash particles, which is an important reason for the dense internal structure. 

**Figure 13:**
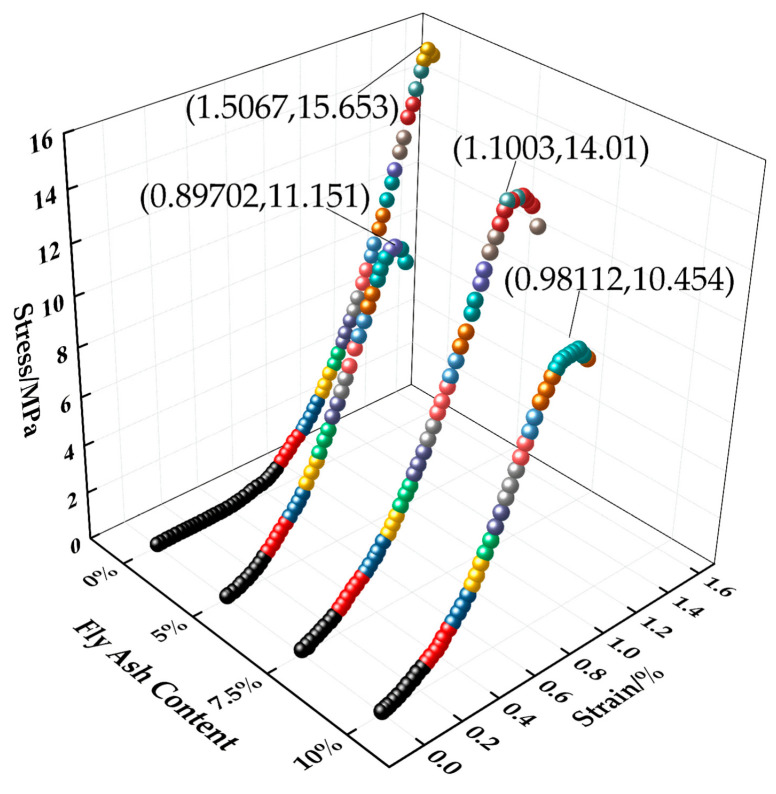
Stress–strain relationship curve of the test block under different fly ash content values.

Figure 13 shows the stress–strain curve of the test block under different fly ash content values. As can be seen from Figure 13, the specimen without fly ash added has the highest strength, with a peak stress increase of 1.643 MPa compared to that of the specimen with 7.5% doping, and at the same value of stress, it has a larger value of strain compared to that of the specimen with fly ash added. When the fly ash content is 7.5%, the strength of the test blocks is more significant than that of the two types of test blocks with a fly ash content of 5% and 10%, and the peak stress is increased by 2.895 MPa and 3.556 MPa, respectively. The development trend of the three strains is similar. The main reason for the difference in strength caused by the incorporation of fly ash is that fly ash has a certain filling effect inside the test block, which is prominent in improving the structural density of cement slurry. The fluidity of permeable concrete prepared with cement alone is poor, and it is not suitable for large-scale preparation in engineering applications. Under the test conditions, the optimum blending ratio of fly ash is 7.5%.

### 3.3. Influence of Aggregate Particle Grade on the Performance of Permeable Concrete

**Figure 14:**
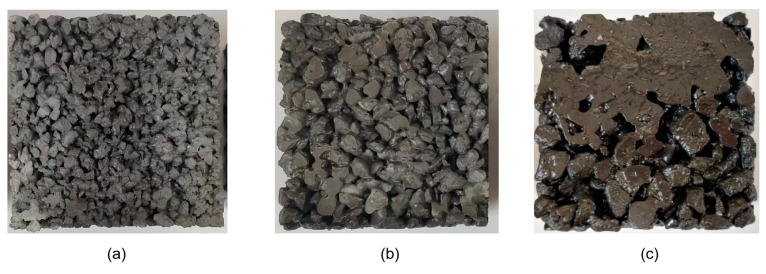
Photos of the bottom of the permeable concrete surface with different aggregate sizes: (**a**) 3–5 mm; (**b**) 5–10 mm; and (**c**) 10–15 mm.

Figure 14 shows photos of permeable concrete with different aggregate particle gradations. As can be seen in Figure 14, under the same shaking time, the 3–5 mm test block does not appear to undergo the slurry sealing phenomenon, and the overall compactness is high. The 5–10 mm test block does not appear to undergo the slurry sealing phenomenon either, but it has obvious pores and a wide distribution of pores. The 10–15 mm test block has an obvious sealing phenomenon, presenting large pores and a low density of aggregate accumulation.

**Figure 15:**
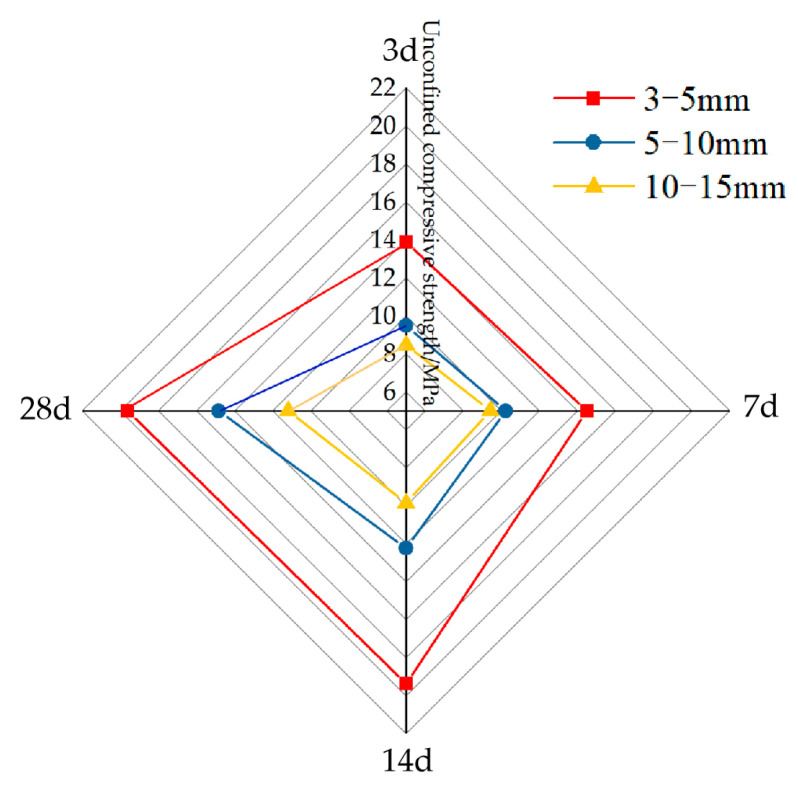
Strength of permeability concrete specimens with different aggregate sizes at different curing ages.

Figure 15 shows the variation law of permeable concrete strength with different aggregate particle gradations and curing ages. In Figure 15, with the increase in curing time, the strength values of the permeable concrete test blocks with different aggregate particle gradations all show an upward trend. When the aggregate particle gradation is 3–5 mm, the strength values of the test blocks are the highest, followed by 5–10 mm, and the lowest values are for 10–15 mm. 

**Figure 16:**
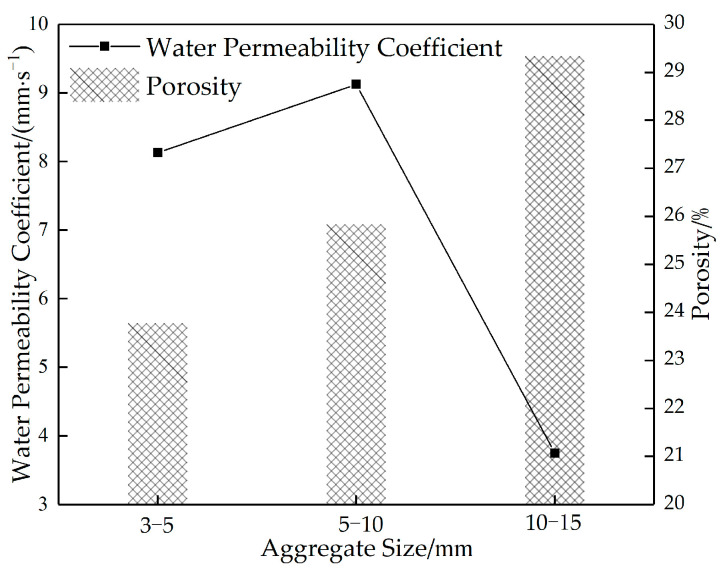
Plot of porosity versus water permeability coefficient of permeable concrete specimens with different aggregate particle sizes.

Figure 16 shows the correlation curve between the water permeability coefficient and the porosity of permeable concrete with different aggregate particle sizes. As can be seen in Figure 16, the porosity increases with the increase in aggregate particle size. The water permeability coefficient increases first and then decreases. When the aggregate particle size is 3–5 mm, the internal structure of the test block is compact, the slurry encapsulation efficiency is high, the slurry sealing phenomenon does not easily appear at the bottom, and the compressive strength is at its maximum. 

When the aggregate particle size is 5–10 mm, the internal structure of the test block is relatively dense, with good strength and water permeability. When the aggregate particle gradation is 10–15 mm, due to its large size, low packing density, easy dislocation between aggregates under pressure, and a large part of the cement slurry existing in the void, this results in the low utilization rate of the cement slurry. During shaking and molding, the cement slurry that exists in the void rapidly sinks due to the action of gravity, resulting in a prominent bottom sealing phenomenon and low water permeability coefficient. However, when the side is used as the top surface of the pressure, the strength increases. Therefore, from the comprehensive consideration of strength and water permeability, the optimal aggregate particle size is 5–10 mm.

### 3.4. Influence of Cut Glass Fiber on Permeable Concrete Performance 

#### 3.4.1. Influence of Fiber Length on the Mechanical Properties of Permeable Concrete

**Figure 17:**
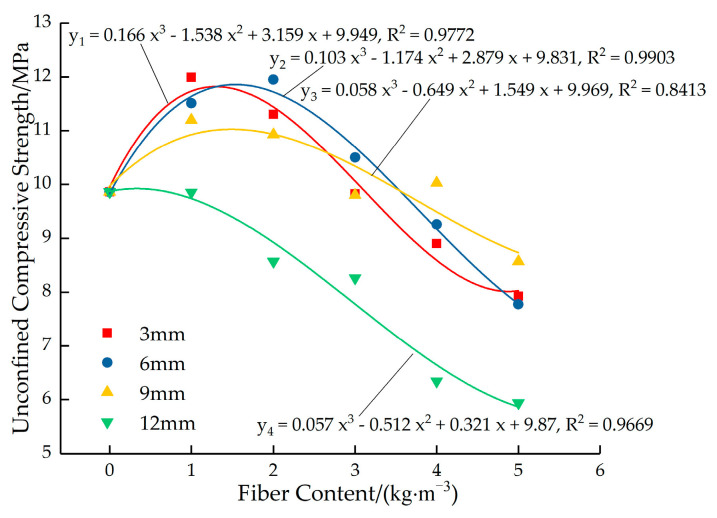
Fitted curves for the variation in the peak strength of permeable concrete with fiber content for different fiber lengths (7 days).

Figure 17 shows the fitted curves for the variation in the peak strength of permeable concrete with fiber dosage for different fiber lengths. As can be seen in Figure 17, when the fiber length is 3 mm and 6 mm, the strength is higher at low content, and the strength decreases rapidly with the increase in fiber content. Moreover, the overall strength of the test block with a fiber length of 6 mm is higher than that with a fiber length of 3 mm. When the fiber length is 9 mm, the strength of the test block decreases slightly with the increase in the dosage, and the overall strength is not high. When the fiber length is 12 mm, the strength of the test block decreases greatly as the fiber content increases and the overall strength is the lowest. 

**Figure 18:**
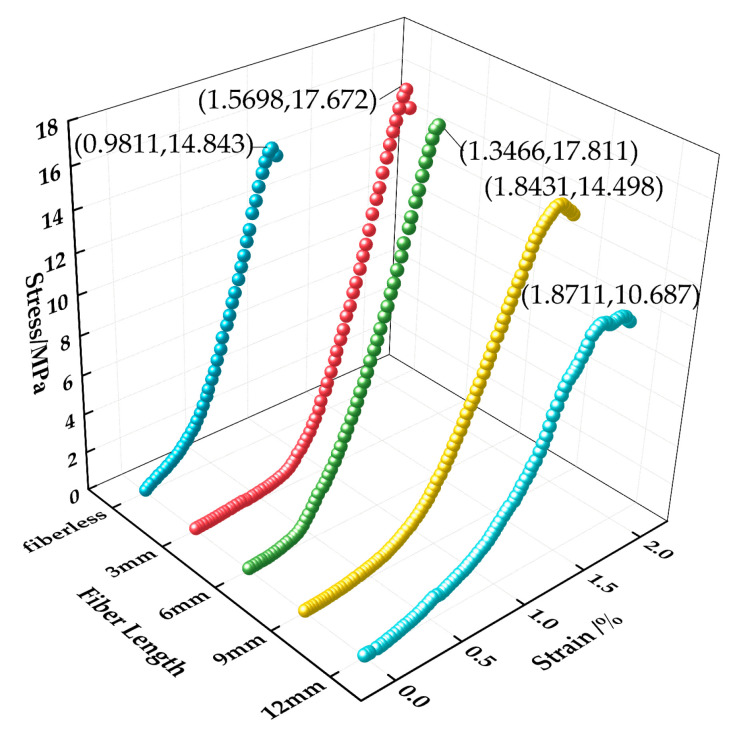
Stress–strain relationship curves of permeable concrete with different fiber lengths (1 kg/m^3^).

**Figure 19:**
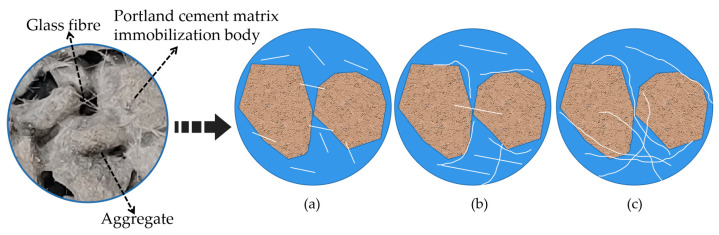
Diagram of different lengths of fibers acting inside the test block: (**a**) 3 mm, (**b**) 6 mm, (**c**) 9 mm, and 12 mm.

Figure 18 shows the stress–strain relationship curves of permeable concrete with fibers of different lengths incorporated at 28 days of curing age. Figure 19 shows the state of fibers of different lengths inside the test block. The figure was drawn by us through the test phenomenon and combined with our own analysis. As can be seen in Figure 18, the peak stress and strain corresponding to different fiber lengths are significantly different, and the changes can be seen in Table 6. The peak stress lifting rate reaches its maximum value at 6 mm, showing a trend of first rising and then decreasing with the increase in fiber length, and the lifting effect of strain shows an overall upward trend. The above phenomenon is due to the fact that the fiber enhances compressive toughness and bearing capacity by restricting the development of cracks when bearing loads. However, the contact area and binding force between the fiber and the matrix are small because the fiber is too short, which makes it easy for the fiber to be pulled out and unable to play an effective pulling force, and the short fiber is not easy to gather in the interior, as shown in Figure 19a. The fiber resistance to strain is small under pressure. Excessive fiber length will affect the bonding effect of the internal cement base. Long fibers are easy to interleave and aggregate, resulting in the “clumping” of fibers inside the test block during mixing, as Figure 19c shows, which affects the combination of slurry and aggregate, resulting in the low strength of the test block, but the fiber plays a “buffering role” during compression, so its strain is larger [32].

#### 3.4.2. Influence of Fiber Length on the Permeable Performance of Permeable Concrete

**Figure 20:**
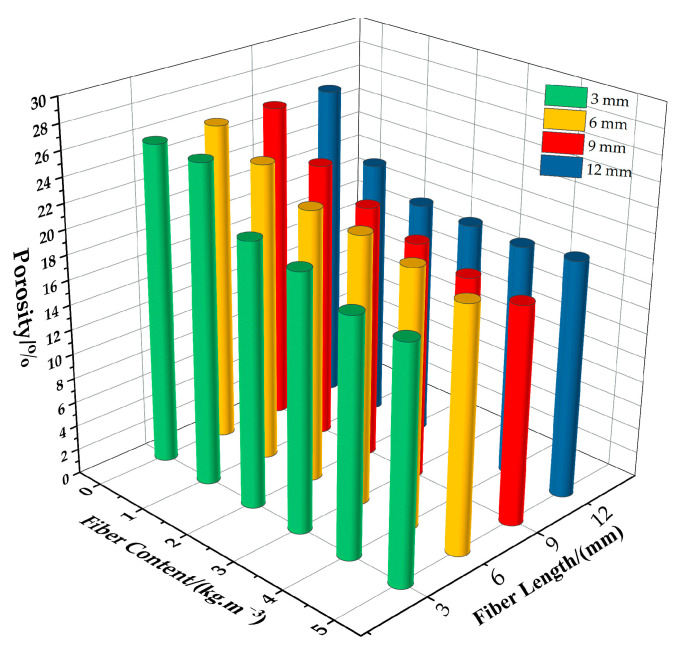
Relationship between changes in the porosity of permeable concrete and fiber dosage for different fiber lengths.

**Figure 21:**
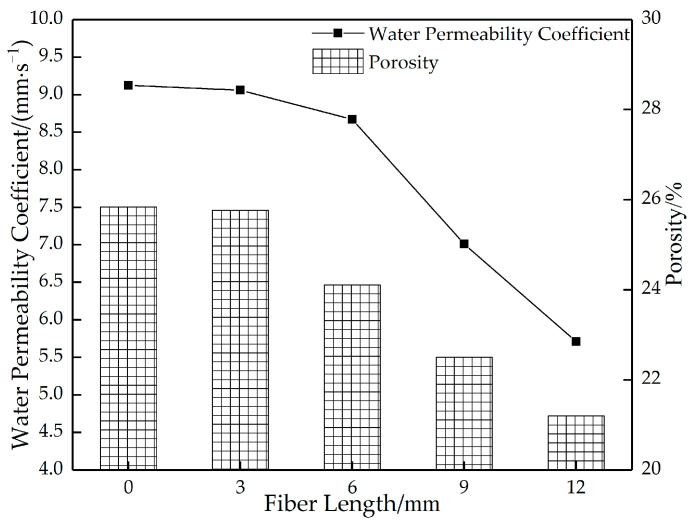
Relationship between the porosity and water permeability coefficient of permeable concrete with different fiber lengths (2 kg/m^3^).

Figure 20 shows the relationship between changes in the porosity of permeable concrete and fiber dosage for different fiber lengths. Figure 21 shows the relationship between porosity and the water permeability coefficient of permeable concrete with different fiber lengths (1 kg/m^3^). As can be seen in Figure 20, with the increase in fiber content and parameter length, the porosity shows a decreasing trend. The longer the fiber, the more obvious the trend. This is because the longer the fiber is, the more connected pores are occupied, resulting in low porosity. The longer the fiber is, the more likely it is to bend and cause clumping when stirred, affecting the porosity. As can be seen in Figure 21, when the content is constant, the fiber length increases, and the porosity and water permeability coefficient both decrease. This is because the incorporation of fibers affects the effective interconnecting pores in the interior. When the fibers are short, the fibers are dispersed more evenly in the interior, which has little influence on the porosity and water permeability coefficient. When the fiber is longer, the fiber occupies more pores in the interior and blocks most of the connected pores, which reduces the porosity and water permeability coefficient significantly.

#### 3.4.3. Influence of Fiber Content on the Mechanical Properties of Permeable Concrete

**Figure 22:**
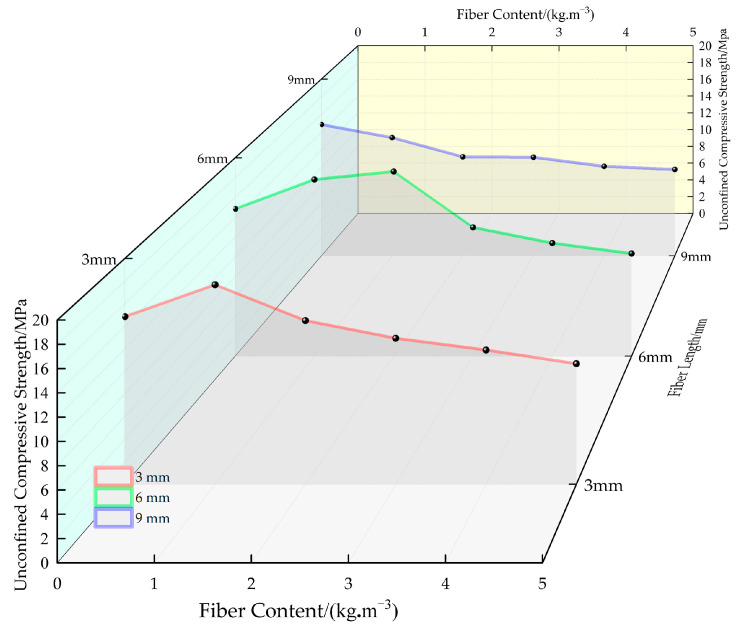
Variation curves of the strength of permeable concrete with different fiber content values (28 days).

**Figure 23:**
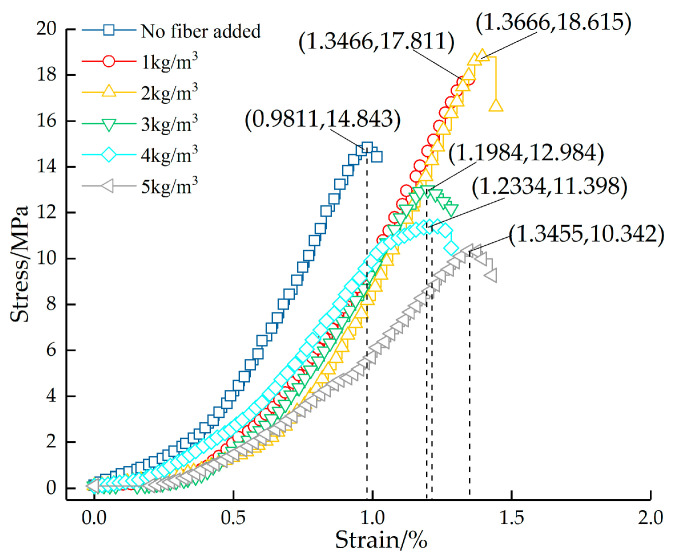
Stress–strain relationship of permeable concrete with different fiber content values (6 mm).

Figure 22 shows the variation curves of the strength of permeable concrete with different fiber content values. Figure 23 shows the stress–strain relationship of permeable concrete with different fiber content values. As can be seen in Figure 22, the overall strength value of the test block is high when the fiber content is low, but the strength value shows a decreasing trend as the fiber content increases. As can be seen in Figure 23, when the fiber length is 6 mm, the strength value first increases and then decreases with the increase in fiber content. The peak stress is 18.615 MPa when the fiber content is 2 kg/m^3^, which is 25.41% higher than that of the test block without fiber. When the fiber content is 5 kg/m^3^, the peak stress is 10.342 MPa, which is 30.32% lower than that of the test block without fiber. The reason is that the increase in fiber content affects the bonding force of the internal cemented layer, and the uneven dispersion causes fiber aggregation; however, this increases the deformation effect and improves the compressive toughness. Therefore, the strength of the test block is low, but the strain value is large at high content.

#### 3.4.4. Influence of Fiber Content on the Permeable Performance of Permeable Concrete

**Figure 24:**
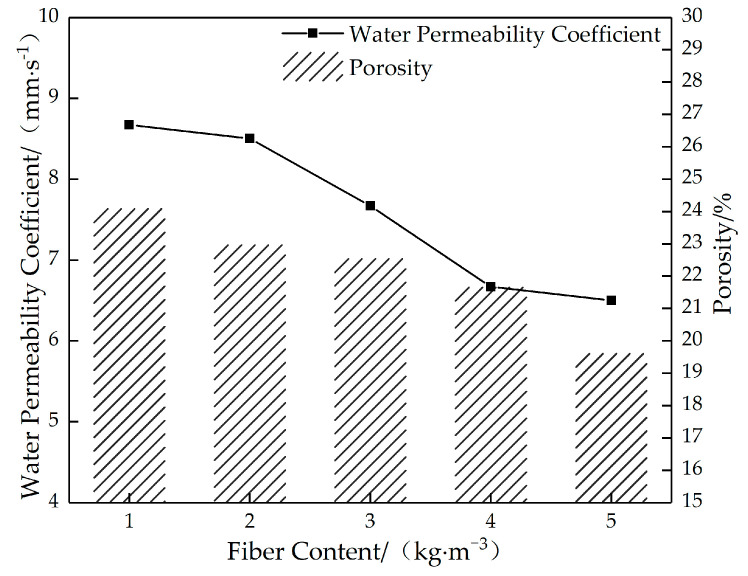
The relationship between the porosity and water permeability coefficient of permeable concrete with different fiber content values (6 mm).

**Figure 25:**
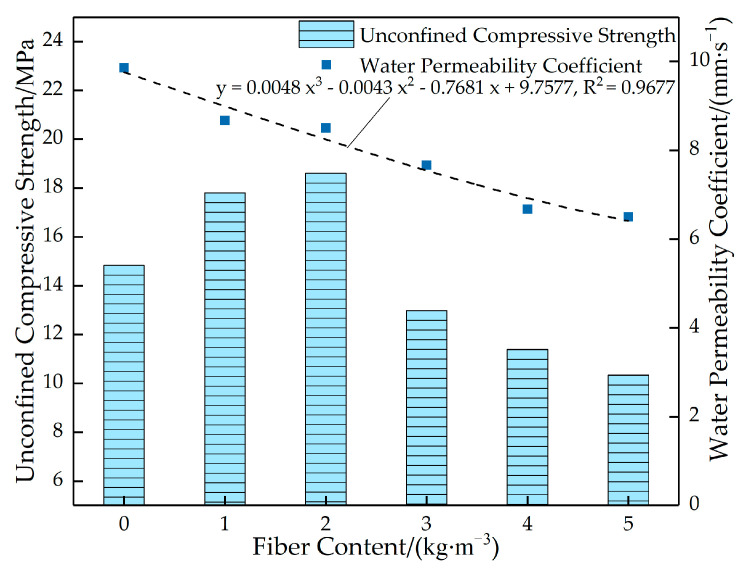
Relationship between the strength and water permeability coefficient of permeable concrete with different fiber content values (6 mm).

Figure 24 shows the relationship between the porosity and water permeability coefficient of permeable concrete with different fiber content values. Figure 25 shows the relationship between the strength and the water permeability coefficient of permeable concrete with different fiber content values. As can be seen in Figure 24, the porosity and water permeability coefficient gradually decrease with the fiber content. This is because the addition of glass fiber affects the size of internal pores, resulting in a decrease in the volume of connected pores of permeable concrete and an increase in the number of closed pores in the test block, thus presenting a decrease in effective porosity. As can be seen in Figure 25, with the increase in fiber content, the strength value first increases and then decreases. When the fiber content is 2 kg/m^3^, the strength reaches the maximum value, and the water permeability coefficient remains at a high level. The reason is that in a certain fiber volume content range, the increase in fiber content will cause the phenomenon of “clumping” and plugging the pores; when the effective porosity is reduced, the water permeability coefficient will be reduced.

### 3.5. Strain Cloud Image Analysis Based on Vic-3D Technology

**Figure 26:**
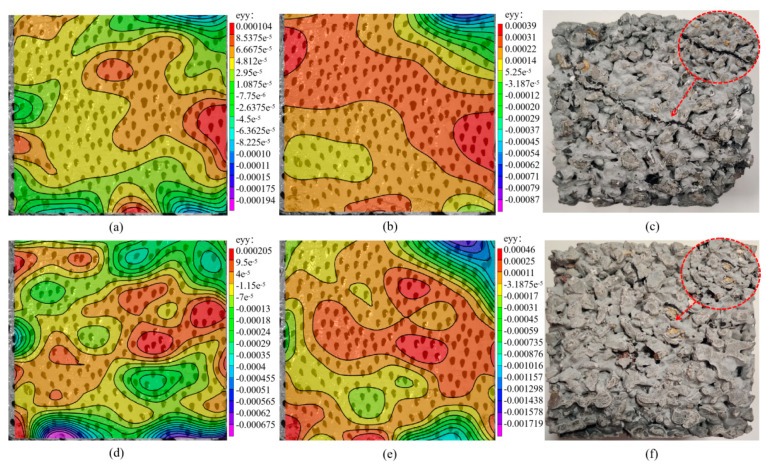
Vertical strain cloud map of permeable concrete based on Vic-3D technology: (**a**) ordinary permeable concrete precompression stage, (**b**) ordinary permeable concrete after compression, (**c**) ordinary permeable concrete damage photos, (**d**) reinforced permeable concrete at the precompression stage, (**e**) reinforced permeable concrete after compression, and (**f**) reinforced permeable concrete damage photos.

In order to study the failure law of permeable concrete, Vic-3D technology was used to study the vertical strain of permeable concrete during compression. Figure 26a,b shows the vertical strain cloud diagram of the front facade of the permeable concrete test block without glass fiber in the loading process, and (c) is the front facade diagram of the actual test block under compression failure. Figure 26d,e demonstrates the vertical strain cloud diagram of the front facade of the reinforced permeable concrete test block mixed with glass fiber during the loading process, and (f) is the front facade diagram of the actual compressive failure of the reinforced test block. It can be seen in Figure 26a–c that the permeable concrete specimens without glass fibers showed strain concentration from the edge of the front elevation of the specimens at the early stage of loading, presenting a tendency to extend from the edge to the interior with the loading process, and presents an inclined strain concentration area in the late stage of loading, showing an obvious failure trend. This is because permeable concrete without fiber mainly relies on the internal cement matrix to resist the deformation caused by pressure, so its brittle characteristics are prominent. When cracks occur, they will be destroyed with the development of the cracks. According to Figure 26d–f, it can be seen that the stress concentration area of the reinforced permeable concrete test block at the early stage of compression is generated from the inside and then diffused around, and the vertical strain value is larger at the same loading stage. This is because the addition of fibers makes the test block subject to a certain “pull” when resisting failure; the test block shows good integrity, the crack develops slowly under pressure and withstands large deformation, and the ductile failure characteristics are prominent.

## 4. Analysis of Fiber Reinforcement Mechanism 

### 4.1. Microscopic Analysis of Glass Fiber in the Cement Matrix

**Figure 27:**
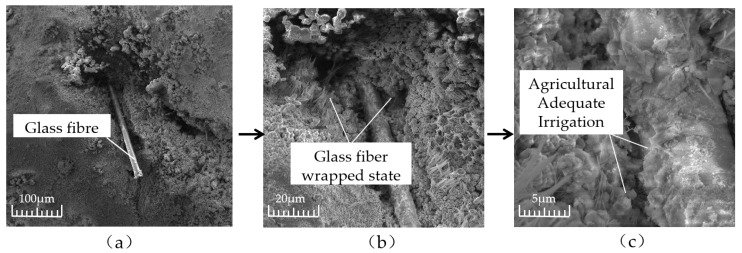
Microstructure of glass fiber in the cementing layer: (**a**) 100 μm; (**b**) 50 μm; and (**c**) 5 μm.

**Figure 28:**
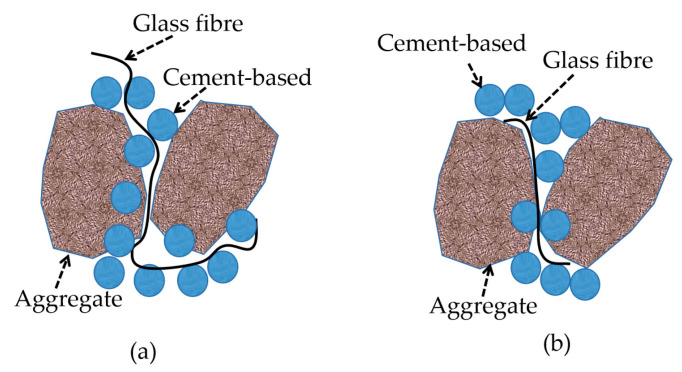
Reinforcement diagram of glass fiber in the cementing layer: (**a**) longer fibers and (**b**) appropriate length fibers.

In order to explore the reinforcement mechanism of fiber in a cement matrix, the wrapping condition of fiber in a cement matrix was analyzed by a scanning electron microscope. Figure 27 shows the wrapping state of glass fiber in the cementation layer inside the permeable concrete. Figure 27 indicates that the bonding effect between the glass fiber and cement matrix is good. The slurry tightly wraps the fiber, and the hydration product of cement adheres to the fiber. Therefore, when the cement layer is destroyed, the fiber can effectively inhibit it. When the fiber is added to permeable concrete, the bonding performance between aggregates is enhanced. When the fiber is longer, it may cling up and bend during the mixing process, which affects the bonding force between the cement and aggregate, increases the void inside the cement slurry, and forms a weak surface, thus affecting the reinforcement effect, as shown in Figure 28a. When the fiber length is appropriate, the bridged fiber between aggregates is not prone to bending, and the fiber of the appropriate length can effectively inhibit crack development and improve the compressive capacity, as shown in Figure 28b. However, the strength enhancement effect of fiber on permeable concrete depends on the strength of the slurry coated with aggregate on the one hand and the content of fiber on the other hand. Research shows that the greater the fiber content, the better the strength effect [33].

### 4.2. Fiber Reinforcement Factor Theory

In order to further explore the influence of glass fiber on permeable concrete, the strength reinforcement factor *α* influenced by the fiber is introduced. Reinforcement factor α is the efficiency of fiber content to improve the peak strength of the test block. The reinforcement factor α is calculated according to Equation (4):

(4) $$\alpha =\frac{f_{\lambda }}{f_{0}}-1$$

where α is the fiber reinforcement factor, $f_{\lambda }$ is the peak strength of fiber permeable concrete, and $f_{0}$ is the peak strength of the test block without adding fiber.

**Figure 29:**
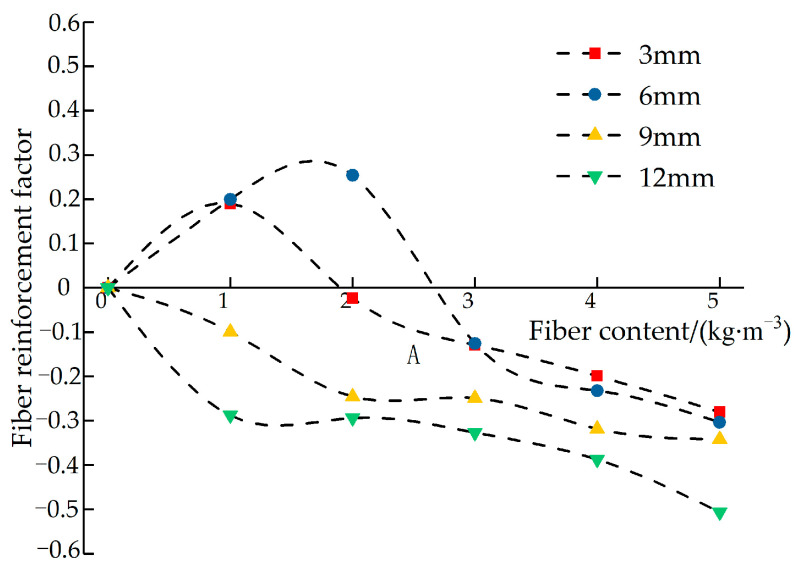
The relationship between the reinforcement factors of different fiber lengths and dosages.

Figure 29 shows the strength reinforcement factor curve influenced by the fiber length. It can be seen in Figure 29 that the reinforcement effect of different fiber lengths on the test block is quite different: when the length is 3 mm, the reinforcement effect is better at a low dosage, and the reinforcement factor decreases slowly with the increase in dosage. When the fiber length is 6 mm, the reinforcement effect is obvious, and the decline rate is slow at a low dosage. The fibers with lengths of 9 mm and 12 mm have an obvious negative effect on the reinforcement of the test block. When the fiber length is too long, the fiber dispersion in the test block is poor, and the fiber aggregation becomes more prominent with the increase in the dosage, so it cannot play an effective strengthening role. When the fiber length is too short, its dispersion is good, and it can play a certain strengthening role at low content, but it is more likely to be pulled out when the internal cemented layer is damaged by the force, so the reinforcement degree is not high. With the increase in content, the reinforcement effect is also reduced due to the uneven dispersion of the fiber.

According to the concept of “crowding factor” [34], when the amount of fiber incorporation is constant, the shorter the glass fiber incorporation is, the larger the volume fraction will be, so the rotation dislocation of the fiber is suppressed, and the contact between the fiber and the cement matrix is more continuous. Alkaline conditions have a certain influence on the glass fiber. The Ca(OH)_2_ generated by cement hydration reacts chemically with SiO_2_ in the glass fiber, weakening the structural relationship between the fibers and causing deterioration of their mechanical properties.

## 5. Conclusions

(1) The shaking time has a significant influence on the strength and water permeability of permeable concrete. With the extension of shaking time, the strength of the test block gradually increases, and the water permeability coefficient gradually decreases. When the shaking time is 3 s, the performance of the test block is the most balanced; that is, it has high strength while satisfying good water permeability.
(2) Fly ash can enhance structural compactness and improve slurry fluidity. When the fly ash content is 7.5%, the permeable concrete has high strength while ensuring good workability.
(3) With the increase in aggregate particle composition, the porosity of permeable concrete showed an increasing trend, and the water permeability coefficient first increased and then decreased, and the strength relationship was 3–5 mm > 5–10 mm > 10–15 mm. The strength and water permeability of the test block can be maintained at a good level when the aggregate particle size is 5–10 mm.
(4) The porosity and water permeability coefficient of permeable concrete is degraded when glass fiber of different lengths and dosages is added. With an increase in glass fiber length, the compressive strength of permeable concrete first increases and then decreases. With an increase in glass fiber content, the changing trend of the compressive strength of permeable concrete is closely related to the fiber length. Under the test conditions, the optimal fiber incorporation length is 6 mm, and the content is 2 kg/m^3^.
(5) Glass fiber was mixed into the permeable concrete, and the fiber network was formed through the bonding effect of cement slurry so as to strengthen the reinforcement effect. The reasonable incorporation of fiber can not only have a better permeable effect but also improve strength and slow down the development of cracks, which has a certain value for the application of road engineering.

## Figures and Tables

**Table 1:** Physical properties of Portland cement.

Specific Surface Area (m^2^/kg)	Setting Time (min)		Rupture Strength (MPa)		Compressive Strength (MPa)	
	Initial Set	Final Set	3d	28d	3d	28d
351	168	258	5.5	9.1	23.9	50.1

**Table 2:** Performance index of fly ash.

Density (g/cm^3^)	Burn Loss	Water Content	Activity Index	Stability
2.85	≤4.5%	≤1.0%	80%	on test

**Table 3:** Performance index of crushed stone aggregate.

Particle Size (mm)	Performance Density (kg/m^3^)	Bulk Density (kg/m^3^)	Void Volume Fraction
3~5	1570	2780	43.52%
5~10	1483	3026	50.99%
10~15	1600	2810	43.06%

**Table 4:** Mix ratio design of ordinary permeable concrete.

Particle Size (mm)	Aggregate Dosage (kg/m^3^)	Cement Content (kg/m^3^)	Fly ash Consumption (kg/m^3^)
3~5	1453.56	482.38	25.38
5~10	1538.61	371.92	19.58
		352.92	39.15
		362.25	29.25
10~15	1568.01	365.17	19.22

**Table 5:** Test scheme and mix ratio of glass fiber permeable concrete.

Particle Size (mm)	Cement Content (kg/m^3^)	Fly Ash Content (kg/m^3^)	Fiber Length (mm)	Fiber Content (kg/m^3^)
5~10	362.25	29.25	3 mm	1, 2, 3, 4, 5
5~10	362.25	29.25	6 mm	1, 2, 3, 4, 5
5~10	362.25	29.25	9 mm	1, 2, 3, 4, 5
5~10	362.25	29.25	12 mm	1, 2, 3, 4, 5

**Table 6:** Peak value and growth rate of the stress–strain curve.

Fiber Length (mm)	σ_max_ (MPa)	Rate of Increase	ε	Rate of Increase
3	17.672	19.06%	1.5689%	59.91%
6	17.811	20.00%	1.3466%	37.25%
9	14.498	−2.32%	1.8431%	87.86%
12	10.687	−28.00%	1.8711%	90.71%

## Data Availability

The data used to support the findings of this study are available from the corresponding author upon request.
